# Contemporary and Future Perspectives on Thoracic Trauma Care: Surgical Stabilization, Multidisciplinary Approaches, and the Role of Artificial Intelligence

**DOI:** 10.3390/jcm14228041

**Published:** 2025-11-13

**Authors:** Chiara Angeletti, Gino Zaccagna, Maurizio Vaccarili, Giulia Salve, Andrea De Vico, Alessandra Ciccozzi, Duilio Divisi

**Affiliations:** 1 Anesthesiology, Intensive Care and Pain Medicine, “Giuseppe Mazzini” Hospital of Teramo, 64100 Teramo, Italy; 2 Thoracic Surgery Unit, “Giuseppe Mazzini” Hospital of Teramo, 64100 Teramo, Italy; gino.zaccagna@aslteramo.it (G.Z.); duilio.divisi@aslteramo.it (D.D.); 3 Department of Life, Health and Environmental Sciences (MeSVA), University of L’Aquila, 67100 L’Aquila, Italy

**Keywords:** thoracic trauma, rib fractures, flail chest, surgical stabilization, regional anesthesia, artificial intelligence

## Abstract

Background/Objectives: Thoracic trauma remains a leading cause of trauma-related illness and death. Despite advances in imaging, ventilation strategies, and surgical fixation, its management remains a topic of debate, with varying practices across hospitals. Current Gaps: Although surgical stabilization of rib fractures (SSRF) has shown a mortality benefit in cases of flail chest and in elderly patients, its indications for non-flail cases remain uncertain. Analgesia strategies are evolving, and epidural remains the gold standard; however, it is limited by contraindications. In contrast, regional blocks, such as the erector spinae plane block (ESPB) and serratus anterior plane block (SAPB), are emerging as safer alternatives to opioid and thoracic epidural analgesia (TEA). Artificial intelligence (AI) is transforming imaging interpretation and risk stratification; however, its integration into daily trauma care is still in its early stages of development. Perspective: This article examines the integration of surgical innovation, regional anesthesia, and AI-powered diagnostics as integral components of future thoracic trauma care. We emphasize the importance of standardized surgical criteria, multimodal pain management approaches, and AI-assisted decision-making tools. Conclusions: Thoracic trauma care is shifting toward a personalized, multidisciplinary, and technology-enhanced approach. Incorporating evidence-based SSRF, advanced pain management techniques, and AI-supported imaging can help reduce mortality, enhance recovery, and optimize resource utilization.

## 1. Introduction

Thoracic trauma represents a major global health burden, accounting for nearly 25% of trauma-related deaths [1]. Blunt mechanisms such as motor vehicle collisions and falls predominate and frequently result in rib fractures, flail chest, pulmonary contusions, and associated vascular or spinal injuries [2]. Flail chest occurs in approximately 7% of patients with blunt chest trauma and carries a mortality rate of 10–20% [3]. It is essential to note that the definition of flail chest differs slightly across various literature sources and clinical guidelines. In this paper, we adopt the widely accepted definition: fractures of three or more consecutive ribs at two or more points, resulting in a segment of the chest wall that becomes mechanically detached from the rest of the thoracic cage (paradoxical chest wall motion). Supportive care remains the cornerstone of management; however, advances in surgical stabilization of rib fractures (SSRF), multimodal analgesia, and artificial intelligence (AI) are transforming the field. Despite these advances, variability persists in surgical indications, timing, analgesic strategies, and imaging workflows [4]. Management approaches have shifted from conservative strategies—focused on pain control with regional anesthesia and respiratory support—to more frequent adoption of surgical fixation, particularly in level I trauma centers with access to modern osteosynthesis devices [5]. SSRF and regional analgesia have reduced the sequelae of flail chest, including prolonged ventilator support and the need for invasive mechanical ventilation. These interventions are now integrated into structured management algorithms designed to minimize complications and ventilator dependence [6].

Unlike sternal fractures, where the surgical indications and techniques to be used are very clear [7,8], indications for rib fixation include prolonged mechanical ventilation, marked chest wall deformity, and pain refractory to conservative measures. National database studies demonstrate that use of SSRF has risen markedly, with a 76% increase in procedures between 2007 and 2014 [9,10]. Compared with nonoperative management, SSRF in flail chest reduces mortality, lowers pneumonia rates, and decreases the need for ventilator support [5]. Mortality among patients with blunt thoracic trauma and rib fractures can range from 10% to 25% [9].

A systematic review of 29 studies further showed that mortality rises with the number of rib fractures and is strongly influenced by pneumonia, pre-existing cardiopulmonary disease, and advanced age [6]. Analgesia remains central to thoracic trauma care. Thoracic epidural anesthesia (TEA) has long been considered the gold standard, but limitations such as contraindications in anticoagulated patients and technical demands restrict its widespread use [11]. Regional blocks, including ESPB and SAPB, have emerged as safe and effective alternatives, facilitated by ultrasound guidance and associated with favorable safety profiles [12]. This Perspective aims to critically examine the challenges and opportunities in thoracic trauma management, integrating evidence from surgery, anesthesia, and artificial intelligence (AI).

## 2. Current State of Management: Old and New Solutions

Supportive care continues to form the foundation of thoracic trauma treatment, encompassing oxygenation, drainage of pleural collections, and mechanical or non-invasive ventilation [13]. Non-invasive ventilation (NIV) has become increasingly used, but its failure remains associated with poor outcomes. Non-invasive ventilation (CPAP, BiPAP) is preferred for patients with borderline respiratory function, as it lowers the risk of pneumonia and ICU stay. A high-flow nasal cannula is a safe alternative, even outside ICU settings. Mechanical ventilation should use protective strategies and is reserved for NIV failure or severe compromise. Postoperatively, early extubation and incentive spirometry support recovery, with long-term pulmonary function often returning to normal after SSRF [14,15]. Predictive scores, such as HACOR and ROX, have been proposed for the early identification of NIV failure, although their validation is limited. Blunt thoracic trauma can cause respiratory failure that requires intubation. A retrospective study of 93 ICU patients examined the HACOR and ROX scores as predictors of non-invasive ventilation (NIV) failure and looked into management strategies. NIV failed in 21.5% of cases, with higher HACOR and lower ROX scores at admission linked to intubation. Alternating helmet CPAP and HFNC reduced failure rates compared to CPAP alone, while epidural analgesia was linked to NIV success versus opioids. These findings suggest HACOR, ROX, ventilation strategy, and analgesia type are key factors in outcomes [16].

### 2.1. Surgical Stabilization of Rib Fractures (SSRF)

Surgical stabilization of rib fractures (SSRF) has emerged as a cornerstone intervention for selected patients. Randomized controlled trials and meta-analyses demonstrate a reduction in mortality and improved pulmonary function, particularly in patients with flail chest and in elderly populations [2,17].

However, evidence remains mixed for patients without flail chest, with one RCT indicating limited benefit [18]. Guidelines from the World Society of Emergency Surgery—Chest Wall Injury Society (WSES–CWIS) recommend early fixation (<72 h) for flail chest and multiple displaced rib fractures [4]. The 2024 WSES–CWIS position paper currently stands as the most comprehensive international consensus on SSRF [4].

- The guidelines synthesize evidence from 287 studies and issue 39 recommendations covering: indications for flail chest, multiple displaced rib fractures, and failure of conservative management.
- Timing: Early intervention (within 72 h) leads to better outcomes.
- Techniques and implants: Various fixation devices are available, with no single method universally superior.

**Figure 1:**
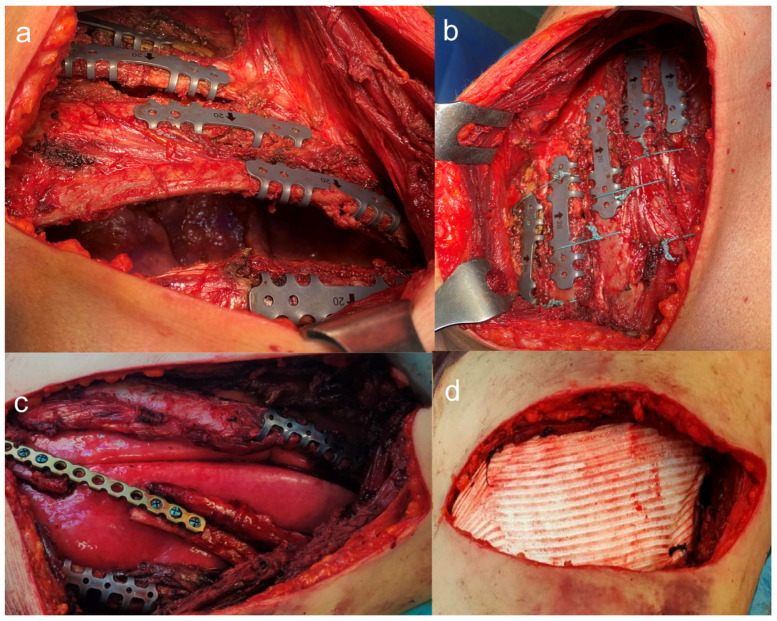
(**a**) Osteosynthesis of a lateral flail chest with struts; (**b**) Rib closure with non-absorbable polyfilament stitches; (**c**) Osteosynthesis of a lateral flail chest using a titanium plate and struts; (**d**) Protection of the lung parenchyma and the chest wall with a PTFE patch.

Benefits include reduced mortality, shorter ICU stays, less reliance on mechanical ventilation, and lower pneumonia rates. Despite these improvements, variations in surgical practices worldwide persist due to differences in expertise, infrastructure, and the adoption of standardized protocols. Recent research highlights the importance of incorporating SSRF into a broader multimodal approach for chest trauma care [18,19]. We retrospectively studied 65 patients [20] to compare surgical fixation methods for flail chest, including plates, Judet’s struts, and wires. Plates and struts improved blood gases faster and enhanced the short-term quality of life better than wires. Wires had shorter surgeries but higher complications and mortality. After a year, the quality of life was similar across groups. Plates and struts were better for early recovery; wires were simpler and less invasive. We also reviewed [21] surgical techniques for chest wall reconstruction in trauma. Titanium plates and Judet’s struts promote rapid respiratory recovery, lower infection rates, and reduce ICU and hospital stays compared to conservative treatment (Figure 1).

Increasingly, intramedullary devices, bioresorbable materials, and meshes (both synthetic and biologic) are used, often together with muscle or omentum flaps, to treat large defects. Vacuum-assisted closure (VAC) lowers the risk of infection. Titanium remains the gold standard for stability; non-rigid mesh provides protection and flexibility. Although surgery is initially costly, it becomes cost-effective due to the improved quality-adjusted life years (QALYs). Conservative management is associated with higher rates of pneumonia, tracheostomy, and ICU stays. Surgical fixation with modern locking plates lowers ventilator days, infections, and enhances long-term quality of life. Alternatives, such as Judet struts and bioabsorbable plates, have limitations, including device breakage and instability. Intramedullary devices are less invasive but carry a risk of migration. Cost analyses demonstrate the advantages of surgery despite higher upfront costs. Future directions include the development of biodegradable materials and the application of 3D printing. Standardized indications and more rigorous trials are necessary. Evidence suggests that surgical stabilization offers benefits over conservative methods, resulting in reduced support requirements, fewer infections, and lower rates of disability. Titanium plates are the most reliable option; wires and bioresorbable materials are less invasive but have limited uses. Reconstruction increasingly employs meshes and biologic materials, supported by VAC therapy and 3D printing for personalized and cost-effective treatment [22]. Hu et al. [23] described a new approach for flail chest using a 3D-printed external fixation guide and VATS. Five patients with rib fractures were successfully treated, showing rapid improvement in breathing, enhanced thoracic stability, and reduced pain. The average surgical duration was 36 min, with minimal blood loss and approximately 85% recovery of thoracic volume. All patients regained normal function within a month, experiencing only minor complications such as superficial wound infections and transient pain. This technique is safe, minimally invasive, cost-effective, and offers a promising alternative to traditional open fixation.

### 2.2. Integrated Diagnostic and Therapeutic Approach for Rib Fractures

#### 2.2.1. Imaging-Based Assessment and Early Diagnostic Stratification

Wong et al. [24], in a pictorial essay, highlight the importance of imaging in thoracic trauma, which accounts for 20–25% of trauma cases and up to 50% of early mortality in severe injuries. The authors propose a systematic interpretation model called “ABC-Please”: A = Air (pneumothorax, pneumomediastinum), B = Bones (rib and thoracic spine fractures, flail chest), C = Cardiovascular (aortic injury, hemothorax, mediastinal hematoma), P = Pulmonary parenchyma and vessels (contusion, laceration, ARDS). Rapid volumetric CT is considered the gold standard for diagnosis, with AI integration suggested to speed up reporting and highlight life-threatening findings. Recent advances in thoracic trauma management combine innovative surgical techniques with advanced imaging strategies. While minimally invasive fixation methods are feasible and cost-effective in selected patients with flail chest, quick CT-based diagnosis and structured interpretation models (e.g., ABC-Please) remain vital for rapidly identifying life-threatening injuries. These developments underscore the dual importance of technological innovation and diagnostic accuracy.

#### 2.2.2. Clinical Decision-Making: Conservative Versus Surgical Stabilization

**Figure 2:**
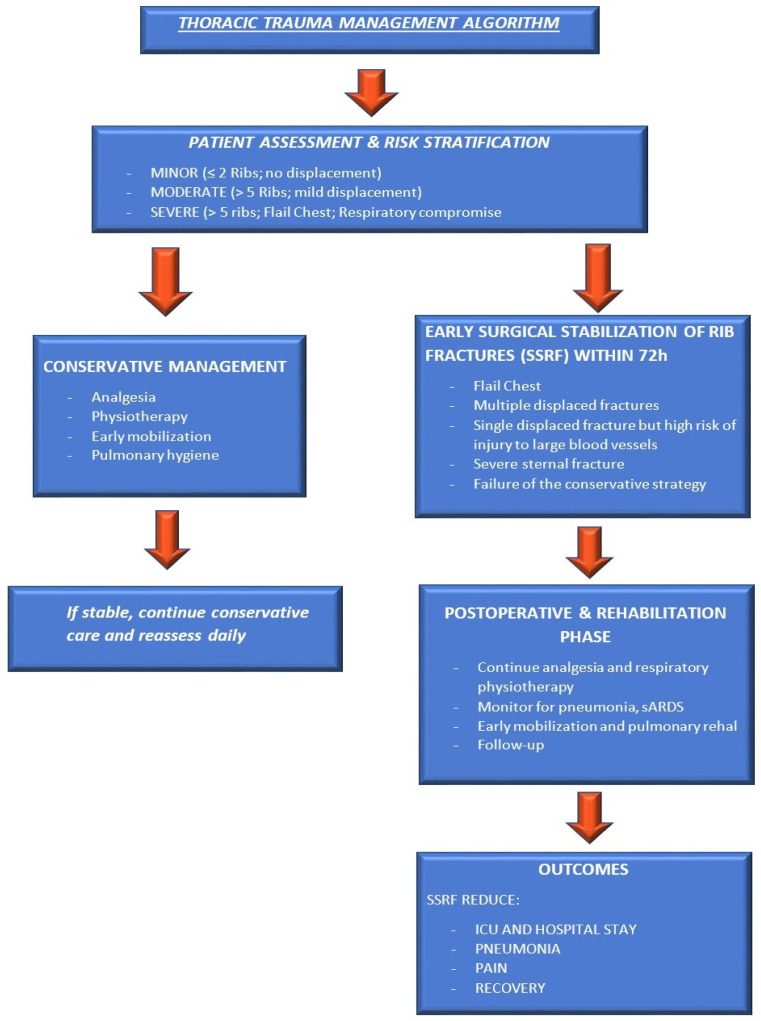
Current thoracic trauma management pathway (supportive care, conservative vs. SSRF, analgesia based on our experience and the literature).

Recent evidence emphasizes SSRF’s importance for high-risk patients with multiple rib fractures. Large database analyses and meta-analyses show SSRF reduces mortality, particularly in flail chest and elderly or dependent groups. Early intervention improves outcomes, but late fixation also benefits survival over conservative treatment, thus improving patient outcomes. Lin et al. [25] conducted a retrospective cohort study using the ACS-TQIP dataset (2020–2022) to evaluate functionally dependent patients with ≥3 rib fractures (AIS ≥ 3). After propensity score matching (294 SSRF vs. 882 controls), SSRF was associated with significantly lower in-hospital mortality (4.8% vs. 8.7%), despite a higher incidence of complications, including unplanned ICU admission, reintubation, and ventilator-associated pneumonia. Early SSRF (≤72 h) improved ventilator days and hospital stay, while late SSRF still offered a survival benefit compared to conservative care. Similar findings are reported by Zhao et al. [17] in a systematic review and meta-analysis that included 47 studies with over 1 million patients. Surgical stabilization of rib fractures (SSRF) was associated with significantly lower mortality (RR, 0.53; 95% CI, 0.39–0.72), especially in patients with flail chest and those over 60 years old. Subgroup analyses revealed that early SSRF (within 72 h) resulted in reduced hospital length of stay and mechanical ventilation duration. However, elderly patients experienced higher rates of pneumonia and longer ICU stays. Overall, SSRF is most effective in cases of flail chest and in elderly patients, particularly when performed early (Figure 2).

### 2.3. Regional Anesthesia in Trauma Chest Pain Management

Rib fractures lead to chronic pain, disability, and a decreased quality of life. In a study of 203 patients, 22% reported chronic pain at six months, which was predictable only by the acute pain at the time of injury [26]. Various methods exist for pain management and form part of the anesthetic strategy for patients undergoing SSRF. These methods focus on the mechanisms of pain detection and transmission related to acute rib fractures.

Peripheral nociceptors are responsible for detecting harmful stimuli in the ribs, joints, connective tissues, and parietal pleura. The intercostal nerves transmit nociceptive information to the dorsal root ganglion of the spinal cord, while the spinothalamic tract carries pain sensations throughout the central nervous system [27]. Understanding the unique pathophysiology and perioperative care for patients with rib fractures is essential for anesthesiologists and surgeons. Besides severe pain, rib fractures impair ventilatory mechanics, reduce cough effectiveness, and increase the risk of atelectasis, pneumonia, respiratory failure, and, in elderly patients, cognitive complications such as delirium. Effective pain management, combined with surgical stabilization in selected cases, is vital to improving outcomes. Various approaches have been explored, including intercostal blocks, epidural anesthesia, thoracic paravertebral block (TPVB), intrapleural infusions, patient-controlled intravenous opioids, and oral medications. Intercostal blocks offer only segmental relief and often require multiple injections, which raises the risk of vascular puncture and pneumothorax [28]. Epidural analgesia, although effective, can cause hypotension and is often contraindicated in trauma patients with coagulopathy, spinal injury, or pelvic instability. In contrast, TPVB involves injecting local anesthetic into the paravertebral space, where spinal nerves, sympathetic fibers, and intercostal vessels are located. The anesthetic spreads along this space, blocking both somatic and sympathetic pathways. Compared to an epidural, TPVB provides better hemodynamic stability and fewer adverse effects, with similar analgesic effects. A recent systematic review of seven RCTs (including 429 patients) compared thoracic epidural analgesia with TPVB after thoracotomy. TEA offered superior early pain relief, while TPVB provided better hemodynamic stability and fewer respiratory complications. Both techniques were similar after 24 h [29]. Recent case reports have confirmed that continuous TPVB is associated with fewer pulmonary complications and lower mortality rates, demonstrating its safety and effectiveness in treating complex trauma [30]. Other fascial or regional techniques are gaining interest, including the erector spinae plane block (ESPB), the serratus anterior plane block (SAPB), the intercostal nerve block (ICNB), and the intertransverse process block (ITPB). ESPB has been shown to provide analgesia comparable to epidural anesthesia in patients with rib fractures, with additional benefits in terms of circulatory stability [31]. ITPB, a newer approach, targets the retro-superior transverse costal ligament, allowing spread into the paravertebral and costotransverse tissue planes [32]. Fascial plane blocks, such as ESPB, SAPB, the pectoral nerve block, Interpectoral and Pectoserratus Plane blocks (IPP + PSP also called PECS block), the transversus thoracic muscle plane block (TTMPB), and parasternal techniques, are increasingly viewed as simple, ultrasound-guided alternatives to neuraxial anesthesia. These blocks enable anesthetic diffusion across multiple nerve levels, providing targeted pain relief for anterior sternotomy, anterolateral incisions, and extensive thoracic pain [33]. Evidence is increasing, although mostly observational, and randomized controlled trials are needed to determine optimal dosing and comparative effectiveness [14]. Among fascial blocks, SAPB has gained popularity as a feasible intervention in the emergency department. A retrospective study of 156 patients showed reduced pain, decreased opioid use, and improved oxygenation indices without complications [34]. The multicenter SABRE randomized trial further confirmed SAPB’s ability to provide faster pain relief and lower opioid requirements within 24 h, although it did not impact pneumonia, ventilation, or mortality [35]. These findings highlight its clinical usefulness as an adjunct, especially for older patients at risk from opioids. Pais et al. [36] also emphasized the feasibility of continuous bilateral SAPB for extensive injuries.

**Figure 3:**
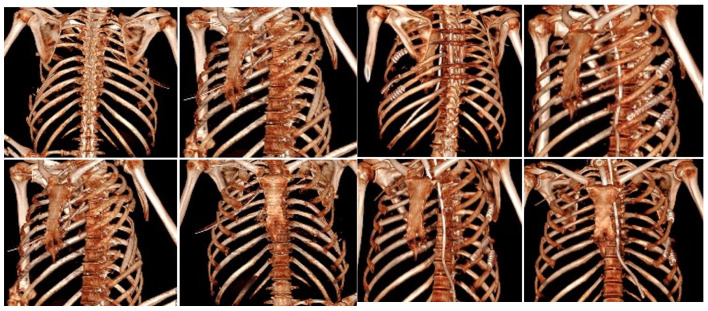
Preoperative CT 3D reconstruction can identify displaced fractures from the third to the eighth left ribs. The patient also experienced a chest wall laceration with lung tissue herniation. The postoperative CT scan shows correction of the fractures through the placement of grafts, titanium plates, and screws.

ESPB has also become a frontline technique due to its simplicity and favorable safety profile, even in patients who are anticoagulated. A scoping review of 37 studies found ESPB reduced pain scores by approximately 40% within 24 h and improved spirometric parameters, with minimal adverse events [37]. It has also facilitated ventilator weaning and supported bilateral analgesia in flail chest [38]. When applied intraoperatively during SSRF, ESPB improved hemodynamic stability, reduced intraoperative opioid consumption, and decreased postoperative analgesic needs [39]. Beyond pain relief, regional anesthesia contributes to better outcomes in elderly trauma patients. In a multicenter cohort of 574 patients with ≥3 rib fractures, catheter-based regional techniques reduced the risk of delirium by 35%, although mortality and respiratory complications were unaffected [40]. While regional anesthesia continues to expand its role, the place of SSRF remains more narrowly defined. Evidence strongly supports surgical stabilization in patients with clinical flail chest requiring ventilation, whereas its extension to non-flail severe injuries remains controversial. A recent RCT found no survival advantage and longer recovery when SSRF was used outside traditional indications [18]. Conversely, regional techniques such as the parascapular sub-iliocostalis plane block (PSIP) have shown promise in the perioperative setting, reducing pain, opioid use, and hemodynamic instability [41]. A recent Bayesian network meta-analysis by Gamberini et al. compared regional anesthesia modalities, concluding that fascial plane blocks offer comparable analgesic efficacy with fewer complications compared to neuraxial techniques [11]. Current evidence supports the use of ultrasound-guided fascial plane blocks, particularly ESPB and SAPB, as effective and safe components of multimodal analgesia for rib fractures. These blocks reliably decrease pain and opioid requirements, are feasible in emergency and perioperative care, and may reduce complications such as delirium in the elderly. In contrast, SSRF should be reserved for patients with a clear flail chest and respiratory compromise until stronger evidence supports its broader use. Future research should prioritize randomized multicenter trials to clarify the comparative roles of ESPB, SAPB, and emerging blocks such as ITPB, with standardized outcomes including pulmonary complications, delirium, and long-term recovery (Figure 3).

## 3. Emerging Role of AI in Thoracic Trauma

Recent research has highlighted the role of artificial intelligence (AI) in thoracic trauma imaging, especially in detecting rib fractures and predicting patient outcomes. Tan et al. [42] showed that a deep learning computer-aided design (CAD) system significantly improved diagnostic sensitivity for rib fractures in patients with blunt chest trauma. In 214 patients, intern sensitivity increased from 68.8% to 91.7%, and radiologists’ sensitivity rose from 86.5% to 94.6%. The system also halved reporting times, increased diagnostic confidence—particularly for occult fractures—and narrowed the gap between junior and senior radiologists. Sun et al. [43] built on these findings in a real-world prospective study comparing AI-assisted radiologists with the traditional double-reading workflow. In 243 consecutive trauma patients undergoing CT scans, AI support increased patient-level sensitivity from 69.2% to 94.2%, while maintaining 100% specificity. Although AI alone demonstrated excellent diagnostic performance, combining AI with radiologists yielded the best results, particularly in identifying recent fractures that are often difficult to detect in initial scans. Besides diagnostic support, machine learning methods have also been used for prognosis. Vazirizadeh et al. [44] used a dataset of 2860 trauma patients to develop models, including Random Forest and XGBoost, achieving an AUC over 0.96 and an accuracy of 0.99 in predicting intrathoracic injuries. Key predictive features included chest pain, dyspnea, Glasgow Coma Scale (GCS), respiratory rate, and oxygen saturation, showing that AI could assist not only in radiological interpretation but also in early triage and risk assessment. Briody et al. [45] offered a broader view in a narrative review summarizing AI applications in acute thoracic imaging. The review highlighted that AI algorithms have consistently enhanced the detection of rib fractures, pneumothoraces, pulmonary contusions, and acute pulmonary embolism, often with sensitivities and specificities exceeding 90%. The authors emphasized AI’s potential to expedite reporting and support decision-making, but also cautioned about its limitations, including false positives, the need for validation across multiple centers, and the risk of exacerbating radiologist burnout if not properly integrated into clinical workflows. Overall, these studies suggest that AI is poised to become a crucial part of thoracic trauma care, enhancing diagnostic accuracy, boosting efficiency, and potentially aiding in prognosis and triage in emergency situations.

## 4. Perspectives

**Figure 4:**
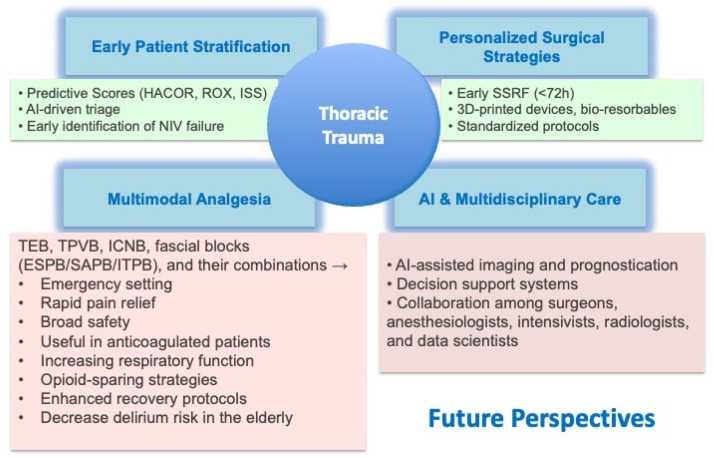
Future perspectives in thoracic trauma care (AI, personalized surgery, multimodal analgesia).

Despite progress, several challenges remain unresolved. Patient selection for SSRF is inconsistent, with significant variability across centers [4]. Chronic pain and disability persist in survivors of rib fractures. A study conducted in Tunisia evaluated the prevalence, associated factors, and psychosocial impact of chronic pain after chest trauma. Among 54 patients interviewed one year after hospitalization, 79.6% reported chronic pain, which was of moderate intensity and with neuropathic characteristics in 90% of cases. About 40% showed symptoms of PTSD. The main areas affected were daily activities, work, sleep, and mood. Chronic pain after chest trauma is therefore common and highly disabling; its prevention requires early identification of at-risk patients, proper acute pain management, and a multidisciplinary approach [46]. Multimodal analgesia strategies are underused, and high-quality randomized evidence is lacking for many regional techniques. Diagnostic workflows remain slow, with chest X-rays missing up to 50% of rib fractures, which can delay interventions [24]. The merging of technological innovation and clinical practice is transforming thoracic trauma care. Early SSRF within 72 h is increasingly recognized as best practice, resulting in reduced ICU stay, ventilator days, and mortality [17,25]. Advances in materials science, including 3D-printed fixation devices and bioresorbable implants, provide opportunities for personalized and less invasive stabilization [20]. Analgesia is also evolving. Fascial plane blocks (ESPB, SAPB, PIFB) are being incorporated into multimodal regimens to decrease opioid use and improve respiratory mechanics. Combined approaches may offer superior pain control while reducing complications. Artificial intelligence is quickly expanding its role in thoracic trauma imaging. AI-driven models improve the detection of rib fractures, enhance diagnostic confidence, and increase interobserver agreement. In real-world studies, AI-assisted radiologists demonstrated greater sensitivity compared to traditional double reading. AI applications also extend to prognosis, predicting ICU admission and ventilator needs using clinical and radiologic data. The future of thoracic trauma care depends on integration. Standardized indications and perioperative protocols for SSRF are urgently needed, supported by multicenter randomized controlled trials. Cost-effectiveness analyses should guide the widespread adoption, including the use of quality-adjusted life years (QALY) metrics. Multimodal analgesia regimens must be optimized and validated in prospective studies, with fascial plane blocks as key components. Although artificial intelligence tools show strong potential to improve triage accuracy, imaging prioritization, and personalized risk assessment, their safe integration into clinical practice requires thorough prospective validation across diverse patient groups and clearly defined regulatory frameworks governing their use. This oversight helps ensure reproducibility, interpretability, and patient safety, preventing premature or inappropriate clinical adoption. Additionally, thoracic trauma management must remain fundamentally multidisciplinary, involving the expertise of surgeons, anesthesiologists, intensivists, radiologists, and data scientists (Figure 4).

## 5. Conclusions

Thoracic trauma persistently presents a significant burden on global health systems. Recent developments in SSRF, regional anesthesia, and artificial intelligence offer promising opportunities for enhancing patient outcomes. By adopting early surgical stabilization, implementing multimodal analgesia, and utilizing AI tools, thoracic trauma management can evolve into a personalized, efficient, and evidence-based approach. Collaboration across multiple centers and innovative research initiatives will be essential to actualize this vision.

## 6. Key Messages

- Thoracic trauma is responsible for 25% of deaths related to injuries.
- SSRF reduces mortality, particularly in patients with flail chest and the elderly.
- Fascial plane blocks (ESPB, SAPB) offer safe and effective alternatives to neuraxial analgesia.
- AI enhances the detection of rib fractures, risk stratification, and prognostication.
- Future models should incorporate early surgery, multimodal pain management, and AI-driven multidisciplinary care.

## Data Availability

No new data were created or analyzed in this study. Data sharing is not applicable to this article.

## Abbreviations

The following abbreviations are used in this manuscript:

SSRF	Surgical Stabilization of Rib Fractures
ESPB	Erector Spinae Plane Block
SAPB	Serratus Anterior Plane Block
TEA	Thoracic Epidural Analgesia
AI	Artificial Intelligence
NIV	Non-invasive ventilation
HFNC	High-Flow Nasal Cannula,
CPAP	Continuous positive airway pressure
BiPAP	stands for Bilevel Positive Airway Pressure.
WSES	World Society of Emergency Surgery
CWIS	Chest Wall Injury Society
VAC	Vacuum-assisted closure
QALYs	Quality-Adjusted Life Years
VATS	Video-assisted thoracoscopic surgery
ICNB	Intercostal Nerve Block
ITPB	Intertransverse Process Block
IPP	Interpectoral Plane block
PSP	Pectoserratus Plane block
TTMPB	Transversus Thoracic Muscle Plane Block
PSIP	Parascapular Sub-Iliocostalis Plane block
RCT	Randomized Controlled Trial
CAD	computer-aided design
CT	computerized tomography scan
GCS	Glasgow Coma Scale
PTSD	Post-traumatic stress disorder
